# *N*′-(3-Fluoro­benzyl­idene)-4-hy­droxy-3-meth­oxy­benzohydrazide methanol monosolvate

**DOI:** 10.1107/S1600536812012196

**Published:** 2012-04-06

**Authors:** Qian-Shou Zong

**Affiliations:** a College of Biology and Chemical Engineering, Jiaxing University, Jiaxing Zhejiang 314001, People’s Republic of China, and School of Pharmaceutical Sciences, Zhejiang University, Hangzhou Zhejiang 310058, People’s Republic of China

## Abstract

In the title compound, C_15_H_13_FN_2_O_3_·CH_3_OH, the dihedral angle between the benzene rings of the benzohydrazone mol­ecule is 5.3 (3)°. The C atom of the meth­oxy group is almost coplanar with its attached ring [deviation = 0.017 (2) Å]. The r.m.s. deviation of the 21 non-H atoms of the hydrazone mol­ecule is 0.106 Å. In the crystal, the components are linked by O_m_—H⋯O_h_, N_h_—H⋯O_m_ and O_h_—H⋯O_h_ (m = methanol and h = hydrazone) hydrogen bonds, forming (001) layers.

## Related literature

For related structures, see: Horkaew *et al.* (2012) ▶; Fun *et al.* (2011 ▶); Zhang (2011 ▶).
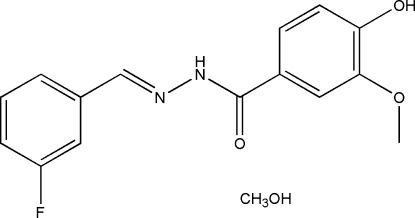


## Experimental

### 

#### Crystal data

C_15_H_13_FN_2_O_3_·CH_4_O
*M* *_r_* = 320.32
Orthorhombic, 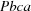

*a* = 14.9566 (18) Å
*b* = 11.1123 (16) Å
*c* = 19.351 (2) Å
*V* = 3216.1 (7) Å^3^
*Z* = 8
Mo *K*α radiation
μ = 0.10 mm^−1^
*T* = 298 K
0.20 × 0.18 × 0.17 mm

#### Data collection

Bruker APEXII CCD diffractometer
Absorption correction: multi-scan (*SADABS*; Sheldrick, 2004 ▶) *T*_min_ = 0.980, *T*_max_ = 0.983
22845 measured reflections
3270 independent reflections
2588 reflections with *I* > 2σ(*I*)
*R*_int_ = 0.030

#### Refinement

*R*[*F* ^2^ > 2σ(*F* ^2^)] = 0.043
*wR*(*F* ^2^) = 0.161
*S* = 1.12
3270 reflections
215 parameters
1 restraint
H atoms treated by a mixture of independent and constrained refinement
Δρ_max_ = 0.28 e Å^−3^
Δρ_min_ = −0.34 e Å^−3^

### 

Data collection: *APEX2* (Bruker, 2004 ▶); cell refinement: *SAINT* (Bruker, 2004 ▶); data reduction: *SAINT*; program(s) used to solve structure: *SHELXS97* (Sheldrick, 2008 ▶); program(s) used to refine structure: *SHELXL97* (Sheldrick, 2008 ▶); molecular graphics: *SHELXTL* (Sheldrick, 2008 ▶); software used to prepare material for publication: *SHELXTL*.

## Supplementary Material

Crystal structure: contains datablock(s) global, I. DOI: 10.1107/S1600536812012196/hb6695sup1.cif

Structure factors: contains datablock(s) I. DOI: 10.1107/S1600536812012196/hb6695Isup2.hkl

Supplementary material file. DOI: 10.1107/S1600536812012196/hb6695Isup3.cml

Additional supplementary materials:  crystallographic information; 3D view; checkCIF report

## Figures and Tables

**Table 1:** Hydrogen-bond geometry (Å, °)

*D*—H⋯*A*	*D*—H	H⋯*A*	*D*⋯*A*	*D*—H⋯*A*
N1—H1⋯O4^i^	0.91 (1)	2.03 (1)	2.916 (2)	164 (2)
O4—H4⋯O3	0.82	1.95	2.772 (2)	176
O1—H1*A*⋯O3^ii^	0.82	1.94	2.7504 (17)	168

Symmetry codes: (i) 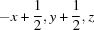; (ii) 
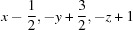
.

**Fig. 1.**
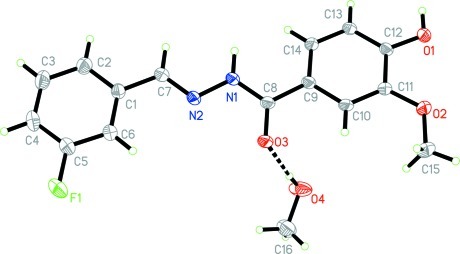
A view of the molecule of the title compound with displacement ellipsoids drawn at the 30% probability level. Hydrogen bond is shown as a dashed line.

**Fig. 2.**
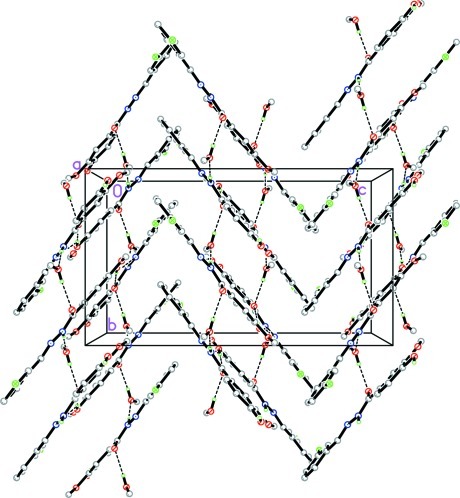
The crystal structure of the title compound, viewed along a axis. Hydrogen bonds are shown as dashed lines.

## supplementary crystallographic information

### Comment

In this paper, the title new benzohydrazide compound, (I), which crystallised as a methanol solvate, is reported.

The dihedral angle between the benzene rings C1–C6 and C9–C14 of the benzohydrazone molecule is 5.3 (3)°. The bond lengths in the benzohydrazone molecule are comparable to those in similar benzohydrazone compounds (Horkaew *et al.*, 2012; Fun *et al.*, 2011; Zhang, 2011). In the crystal, the benzohydrazone molecules are linked by methanol molecules through hydrogen bonds (Table 1), to form layers parallel to the *ab* plane (Fig. 2).

### Experimental

3-Fluorobenzaldehyde (0.124 g, 1 mmol) and 4-hydroxy-3-methoxybenzohydrazide (0.182 g, 1 mmol) were mixed in methanol. The mixture was stirred at room temperature for 1 h to give a colorless solution. Colourless blocks were obtained by slow evaporation from the solution in air.

### Refinement

H1 was located from an electronic map and restrained with N—H distance of 0.90 (1) Å. All other H atoms were placed at calculated positions and refined using a riding model approximation, with C—H = 0.93 or 0.96 Å, O—H = 0.82 Å, and with *U*_iso_(H) = 1.2*U*_eq_(C) or 1.5*U*_eq_(methyl C and O).

### Crystal data

**Table:**

C_15_H_13_FN_2_O_3_·CH_4_O	*D*_x_ = 1.323 Mg m^−^^3^
*M**_r_* = 320.32	Mo *K*α radiation, λ = 0.71073 Å
Orthorhombic, *P**b**c**a*	Cell parameters from 8503 reflections
*a* = 14.9566 (18) Å	θ = 2.5–26.0°
*b* = 11.1123 (16) Å	µ = 0.10 mm^−^^1^
*c* = 19.351 (2) Å	*T* = 298 K
*V* = 3216.1 (7) Å^3^	Block, colorless
*Z* = 8	0.20 × 0.18 × 0.17 mm
*F*(000) = 1344	

### Data collection

**Table:**

Bruker APEXII CCD diffractometer	3270 independent reflections
Radiation source: fine-focus sealed tube	2588 reflections with *I* > 2σ(*I*)
Graphite monochromator	*R*_int_ = 0.030
φ and ω scans	θ_max_ = 26.5°, θ_min_ = 2.1°
Absorption correction: multi-scan (*SADABS*; Sheldrick, 2004)	*h* = −18→17
*T*_min_ = 0.980, *T*_max_ = 0.983	*k* = −13→13
22845 measured reflections	*l* = −24→24

### Refinement

**Table:**

Refinement on *F*^2^	Primary atom site location: structure-invariant direct methods
Least-squares matrix: full	Secondary atom site location: difference Fourier map
*R*[*F*^2^ > 2σ(*F*^2^)] = 0.043	Hydrogen site location: inferred from neighbouring sites
*wR*(*F*^2^) = 0.161	H atoms treated by a mixture of independent and constrained refinement
*S* = 1.12	*w* = 1/[σ^2^(*F*_o_^2^) + (0.0956*P*)^2^ + 0.5869*P*] where *P* = (*F*_o_^2^ + 2*F*_c_^2^)/3
3270 reflections	(Δ/σ)_max_ < 0.001
215 parameters	Δρ_max_ = 0.28 e Å^−^^3^
1 restraint	Δρ_min_ = −0.34 e Å^−^^3^

### Special details

**Table:**

Geometry. All esds (except the esd in the dihedral angle between two l.s. planes) are estimated using the full covariance matrix. The cell esds are taken into account individually in the estimation of esds in distances, angles and torsion angles; correlations between esds in cell parameters are only used when they are defined by crystal symmetry. An approximate (isotropic) treatment of cell esds is used for estimating esds involving l.s. planes.
Refinement. Refinement of F^2^ against ALL reflections. The weighted R-factor wR and goodness of fit S are based on F^2^, conventional R-factors R are based on F, with F set to zero for negative F^2^. The threshold expression of F^2^ > 2sigma(F^2^) is used only for calculating R-factors(gt) etc. and is not relevant to the choice of reflections for refinement. R-factors based on F^2^ are statistically about twice as large as those based on F, and R- factors based on ALL data will be even larger.

### Fractional atomic coordinates and isotropic or equivalent isotropic displacement parameters (Å^2^)

**Table:**

	*x*	*y*	*z*	*U*_iso_*/*U*_eq_	
F1	0.62214 (8)	1.20264 (15)	0.71230 (10)	0.0899 (5)	
N1	0.27077 (9)	0.93828 (13)	0.59540 (8)	0.0396 (4)	
N2	0.33902 (9)	1.00033 (12)	0.62766 (8)	0.0390 (3)	
O1	0.02644 (7)	0.56459 (11)	0.42821 (6)	0.0430 (3)	
H1A	−0.0236	0.5933	0.4332	0.064*	
O2	0.19271 (8)	0.52220 (13)	0.40048 (7)	0.0540 (4)	
O3	0.37143 (7)	0.80593 (11)	0.55390 (8)	0.0497 (4)	
O4	0.39737 (10)	0.57101 (14)	0.59772 (13)	0.0833 (6)	
H4	0.3917	0.6416	0.5862	0.125*	
C1	0.38310 (11)	1.16625 (15)	0.69775 (9)	0.0397 (4)	
C2	0.35539 (13)	1.26065 (17)	0.73975 (10)	0.0500 (5)	
H2	0.2946	1.2747	0.7460	0.060*	
C3	0.41710 (15)	1.33379 (19)	0.77229 (11)	0.0585 (5)	
H3	0.3976	1.3964	0.8004	0.070*	
C4	0.50739 (14)	1.31446 (19)	0.76342 (12)	0.0601 (6)	
H4A	0.5494	1.3629	0.7854	0.072*	
C5	0.53336 (13)	1.22155 (18)	0.72118 (11)	0.0533 (5)	
C6	0.47444 (12)	1.14675 (16)	0.68772 (10)	0.0449 (4)	
H6	0.4947	1.0851	0.6593	0.054*	
C7	0.31604 (11)	1.09122 (16)	0.66320 (9)	0.0419 (4)	
H7	0.2558	1.1105	0.6674	0.050*	
C8	0.29236 (10)	0.84020 (15)	0.55851 (9)	0.0361 (4)	
C9	0.21891 (10)	0.77204 (14)	0.52525 (9)	0.0351 (4)	
C10	0.24240 (10)	0.68253 (15)	0.47763 (9)	0.0383 (4)	
H10	0.3023	0.6698	0.4670	0.046*	
C11	0.17751 (10)	0.61309 (15)	0.44635 (9)	0.0371 (4)	
C12	0.08669 (10)	0.63371 (14)	0.46134 (8)	0.0341 (4)	
C13	0.06435 (10)	0.72117 (16)	0.50909 (9)	0.0381 (4)	
H13	0.0046	0.7337	0.5201	0.046*	
C14	0.12929 (10)	0.79028 (15)	0.54072 (9)	0.0379 (4)	
H14	0.1129	0.8492	0.5724	0.046*	
C15	0.28296 (14)	0.4987 (2)	0.38116 (13)	0.0658 (6)	
H15A	0.3077	0.5685	0.3591	0.099*	
H15B	0.2847	0.4318	0.3498	0.099*	
H15C	0.3173	0.4798	0.4216	0.099*	
C16	0.48620 (15)	0.5484 (2)	0.61393 (16)	0.0715 (7)	
H16A	0.5145	0.6218	0.6283	0.107*	
H16B	0.5164	0.5171	0.5740	0.107*	
H16C	0.4892	0.4906	0.6507	0.107*	
H1	0.2134 (9)	0.965 (2)	0.5972 (14)	0.080*	

### Atomic displacement parameters (Å^2^)

**Table:**

	*U*^11^	*U*^22^	*U*^33^	*U*^12^	*U*^13^	*U*^23^
F1	0.0391 (7)	0.0999 (11)	0.1307 (13)	−0.0092 (7)	−0.0109 (7)	−0.0398 (10)
N1	0.0252 (6)	0.0355 (8)	0.0580 (9)	−0.0004 (5)	−0.0071 (6)	−0.0071 (6)
N2	0.0296 (7)	0.0345 (7)	0.0529 (8)	−0.0032 (5)	−0.0072 (6)	−0.0026 (6)
O1	0.0290 (6)	0.0475 (7)	0.0524 (7)	−0.0020 (5)	−0.0040 (5)	−0.0134 (6)
O2	0.0345 (7)	0.0598 (9)	0.0676 (8)	0.0074 (6)	−0.0031 (6)	−0.0287 (7)
O3	0.0231 (6)	0.0446 (7)	0.0815 (9)	0.0013 (5)	−0.0042 (5)	−0.0166 (6)
O4	0.0364 (8)	0.0443 (9)	0.169 (2)	−0.0031 (6)	−0.0031 (9)	0.0085 (10)
C1	0.0402 (9)	0.0368 (9)	0.0422 (9)	−0.0020 (7)	−0.0044 (7)	−0.0019 (7)
C2	0.0479 (10)	0.0492 (10)	0.0528 (11)	−0.0004 (8)	0.0039 (8)	−0.0106 (9)
C3	0.0659 (13)	0.0514 (12)	0.0583 (12)	−0.0030 (10)	−0.0009 (10)	−0.0193 (9)
C4	0.0610 (13)	0.0533 (12)	0.0659 (13)	−0.0158 (10)	−0.0114 (10)	−0.0157 (10)
C5	0.0407 (10)	0.0546 (12)	0.0646 (12)	−0.0063 (8)	−0.0064 (9)	−0.0086 (10)
C6	0.0410 (9)	0.0414 (9)	0.0524 (10)	−0.0009 (7)	−0.0061 (8)	−0.0082 (8)
C7	0.0318 (8)	0.0421 (9)	0.0519 (10)	0.0004 (7)	−0.0022 (7)	−0.0057 (8)
C8	0.0257 (7)	0.0332 (8)	0.0494 (9)	0.0012 (6)	−0.0016 (6)	0.0000 (7)
C9	0.0266 (7)	0.0331 (8)	0.0456 (9)	−0.0006 (6)	−0.0026 (6)	−0.0007 (7)
C10	0.0241 (7)	0.0397 (9)	0.0511 (9)	0.0034 (6)	−0.0009 (7)	−0.0044 (7)
C11	0.0301 (8)	0.0390 (9)	0.0421 (9)	0.0038 (6)	−0.0014 (6)	−0.0065 (7)
C12	0.0263 (7)	0.0354 (8)	0.0407 (8)	−0.0018 (6)	−0.0038 (6)	−0.0006 (7)
C13	0.0233 (7)	0.0430 (10)	0.0480 (9)	0.0009 (6)	0.0005 (6)	−0.0063 (7)
C14	0.0281 (8)	0.0375 (9)	0.0482 (9)	0.0011 (6)	−0.0001 (6)	−0.0074 (7)
C15	0.0436 (11)	0.0745 (15)	0.0794 (14)	0.0136 (10)	0.0079 (10)	−0.0270 (12)
C16	0.0519 (13)	0.0620 (14)	0.1007 (19)	−0.0047 (10)	−0.0152 (12)	0.0040 (13)

### Geometric parameters (Å, º)

**Table:**

F1—C5	1.355 (2)	C4—H4A	0.9300
N1—C8	1.342 (2)	C5—C6	1.374 (3)
N1—N2	1.3809 (19)	C6—H6	0.9300
N1—H1	0.910 (10)	C7—H7	0.9300
N2—C7	1.269 (2)	C8—C9	1.481 (2)
O1—C12	1.3465 (19)	C9—C14	1.388 (2)
O1—H1A	0.8200	C9—C10	1.401 (2)
O2—C11	1.364 (2)	C10—C11	1.380 (2)
O2—C15	1.425 (2)	C10—H10	0.9300
O3—C8	1.2456 (19)	C11—C12	1.408 (2)
O4—C16	1.388 (3)	C12—C13	1.382 (2)
O4—H4	0.8200	C13—C14	1.381 (2)
C1—C2	1.390 (3)	C13—H13	0.9300
C1—C6	1.397 (2)	C14—H14	0.9300
C1—C7	1.466 (2)	C15—H15A	0.9600
C2—C3	1.382 (3)	C15—H15B	0.9600
C2—H2	0.9300	C15—H15C	0.9600
C3—C4	1.378 (3)	C16—H16A	0.9600
C3—H3	0.9300	C16—H16B	0.9600
C4—C5	1.373 (3)	C16—H16C	0.9600
			
C8—N1—N2	117.91 (13)	C14—C9—C10	119.18 (14)
C8—N1—H1	121.0 (17)	C14—C9—C8	123.21 (15)
N2—N1—H1	121.0 (17)	C10—C9—C8	117.57 (14)
C7—N2—N1	116.24 (14)	C11—C10—C9	120.62 (14)
C12—O1—H1A	109.5	C11—C10—H10	119.7
C11—O2—C15	117.69 (14)	C9—C10—H10	119.7
C16—O4—H4	109.5	O2—C11—C10	125.62 (14)
C2—C1—C6	119.33 (16)	O2—C11—C12	114.55 (14)
C2—C1—C7	119.46 (16)	C10—C11—C12	119.83 (15)
C6—C1—C7	121.18 (16)	O1—C12—C13	123.90 (14)
C3—C2—C1	120.74 (18)	O1—C12—C11	117.05 (14)
C3—C2—H2	119.6	C13—C12—C11	119.04 (14)
C1—C2—H2	119.6	C14—C13—C12	121.15 (14)
C4—C3—C2	120.40 (19)	C14—C13—H13	119.4
C4—C3—H3	119.8	C12—C13—H13	119.4
C2—C3—H3	119.8	C13—C14—C9	120.15 (15)
C5—C4—C3	117.95 (18)	C13—C14—H14	119.9
C5—C4—H4A	121.0	C9—C14—H14	119.9
C3—C4—H4A	121.0	O2—C15—H15A	109.5
F1—C5—C4	117.99 (17)	O2—C15—H15B	109.5
F1—C5—C6	118.35 (18)	H15A—C15—H15B	109.5
C4—C5—C6	123.66 (19)	O2—C15—H15C	109.5
C5—C6—C1	117.91 (17)	H15A—C15—H15C	109.5
C5—C6—H6	121.0	H15B—C15—H15C	109.5
C1—C6—H6	121.0	O4—C16—H16A	109.5
N2—C7—C1	120.97 (15)	O4—C16—H16B	109.5
N2—C7—H7	119.5	H16A—C16—H16B	109.5
C1—C7—H7	119.5	O4—C16—H16C	109.5
O3—C8—N1	120.98 (15)	H16A—C16—H16C	109.5
O3—C8—C9	121.10 (15)	H16B—C16—H16C	109.5
N1—C8—C9	117.89 (13)		

### Hydrogen-bond geometry (Å, º)

**Table:**

*D*—H···*A*	*D*—H	H···*A*	*D*···*A*	*D*—H···*A*
N1—H1···O4^i^	0.91 (1)	2.03 (1)	2.916 (2)	164 (2)
O4—H4···O3	0.82	1.95	2.772 (2)	176
O1—H1*A*···O3^ii^	0.82	1.94	2.7504 (17)	168

Symmetry codes: (i) −*x*+1/2, *y*+1/2, *z*; (ii) *x*−1/2, −*y*+3/2, −*z*+1.
